# Relation of Depression, Anxiety, and Quality of Life with Outcome after Percutaneous Transluminal Coronary Angioplasty

**DOI:** 10.1155/2013/465979

**Published:** 2013-11-10

**Authors:** Suprakash Chaudhury, Kalpana Srivastava

**Affiliations:** ^1^Department of Psychiatry, Pravara Institute of Medical Sciences (Deemed University), Rural Medical College and Hospital, Loni, District Ahmednagar, Maharashtra 413736, India; ^2^Department of Psychiatry, Armed Forces Medical College, Pune, Maharashtra 411040, India

## Abstract

*Background*. Despite, increasing number of percutaneous transluminal coronary angioplasty (PTCA) being performed, there is a paucity of Indian studies on the psychological effects of PTCA. *Aim*. To study the relation of anxiety, depression, and health related quality of life with outcome after PTCA. *Methods*. A total of 35 patients undergoing PTCA were included in the present project with their informed consent. All patients filled a specially designed proforma, the Hospital Anxiety and Depression Scale, Coronary Scale, Seattle Angina Questionnaire, and a health related quality of life measure (EQ 5D) one day before undergoing PTCA. Three days after PTCA patients were reassessed with the Hospital anxiety & depression scale, Seattle angina questionnaire and the EQ 5D. *Results*. Analysis showed that 46% had significant anxiety and 32.1% had significant depression before PTCA. Following successful PTCA, none of the patients had significant anxiety, and only 2 (3.6%) had significant depression. On the Seattle Angina Questionnaire, physical limitation reduced from 67.9 to 48. Disease perception improved from 21.2 to 37.1. On the EQ5D, the health status improved from 42.7 before PTCA to 78.7 after PTCA. *Conclusion*. Successful PTCA resulted in significant reduction in anxiety, depression, and physical limitation and improvement in disease perception and health status.

## 1. Introduction

Coronary heart disease (CHD) remains a major public health problem in industrialized countries because of its major contribution to total mortality. Large international differences in the trends in CHD incidence and mortality have been reported. A number of risk factors for coronary arteriosclerosis, including hyperlipidemia, obesity, hypertension, cigarette smoking, male sex, and diabetes mellitus, have been well documented. Although novel therapeutic strategies are in development to reduce atherogenesis in the coronary arteries, primary or secondary Percutaneous Transluminal Coronary Angioplasty (PTCA) is still the most common method of treatment. Psychiatric disorders are often seen in patients who have undergone PTCA which include depression, anxiety, adjustment disorder, body dysmorphic disorder, substance use, mixed anxiety and depression, sleep disorders, and cognitive disorders. These psychiatric manifestations are predominantly seen in those individuals who are vulnerable or predisposed to certain psychiatric disorders. Fragmented sleep is a problem partly because of psychophysiological symptoms seen usually 1 year after PTCA, with reduced resilience to stress, increasing vulnerability, or diminished coping ability and poorer quality of life. Few studies suggest that persons who responded to their illness by perceiving control over their futures, by having positive expectations about their futures, and by holding a positive view of themselves seemed to be at less risk for a new cardiac event after a first PTCA. In some studies, it was also noted that depression and anxiety constituted independent risk factors for cardiovascular adverse events following myocardial infarction [1–6]. There is a paucity of Indian studies in this field. Specifically there is a lack of studies, which have evaluated anxiety, depression, neuroticism, and quality of life before and after PTCA procedure. In view of the above the present study was undertaken.

## 2. Material and Method

 The study was conducted at the Department of Cardiology of a tertiary care service hospital at Pune, India. The project was approved by the institutional ethical committee. A total of 35 consecutive patients admitted to the hospital for PTCA were included in the study with their informed consent. Patients were interviewed one day prior to the PTCA and three days after the procedure. During the initial interview, demographic and historical data were recorded on a specially designed proforma. All patients underwent a physical examination. Details of cardiac evaluation and laboratory investigations were recorded from case notes. All patients underwent the following psychological tests.

1. *The Hospital Anxiety and Depression Scale* (HADS) [7] is a self-report questionnaire developed to detect adverse anxiety and depressive states in nonpsychiatric departments.
2. *Coronary Scale* [8] was constructed from 8 Eysenck Personality Questionnaire items. It could be used for detecting a high-risk group of emotionally labile subjects.
3. *Seattle Angina Questionnaire* [9] is a 19-item self-administered questionnaire measuring five dimensions of coronary artery disease: physical limitation, anginal stability, anginal frequency, treatment satisfaction, and disease perception.
4. *EQ5D Health Questionnaire* [10] is a widely used generic quality of life scale. It is a standardized instrument for use as a measure of health outcome. The EQ5D comprises five questions on mobility, self-care, pain, usual activities, and psychological status. In addition, there is a visual analogue scale to indicate the general health status with 100 indicating the best health status.

PTCA was performed in patients who had evidence of reversible myocardial ischemia (RMI). RMI was assessed on the basis of the symptom of angina or demonstrable reversibility of ischemia on exercise or pharmacological stress. The modalities used for this included treadmill stress testing, 99 technetium myocardial imaging, or dobutamine stress echocardiography. Only lesions which were hemodynamically significant, that is, greater than 70% reduction in diameter, were addressed. The procedure was performed after informed consent using the femoral access under local anesthesia. Percutaneous balloon angioplasty was done and was followed by stenting as and when indicated. Stents were used in 90% of the angioplasties. After the procedure, the patients were monitored in an appropriate coronary care unit (CCU). The patients were followed up during the immediate post-PTCA period, and any complications were noted from case records. Three days after PTCA, the patients were administered the following psychological tests.

1. Hospital Anxiety and Depression Scale.
2. Seattle Angina Questionnaire.
3. EQ5D Health Questionnaire.

The data, which was collected, was tabulated and statistically analyzed using appropriate psychological tests.

## 3. Results

 A total of 35 patients were admitted for PTCA during the period of study and were included in the study with their consent. The mean (±SD) age of the patients was 60.9 (±10.8) years. The age of the patients ranged from 27 years to 82 years. The majority of the patients were more than 50 years of age, and only two were less than 50 years of age. All the patients were males and either serving or retired military personnels since the study was conducted at a military hospital. All the patients were educated at least up to 10 classes. Mean monthly income of the patients was Rs 6314.3 (±Rs 1430.2). The monthly income of the patients ranged from Rs 5000 to Rs 10,000. The demographic characteristics and the personal habits of the patients undergoing PTCA are given in Table 1. It is seen that the majority of the patients were nonvegetarians, smokers, and nondrinkers. A family history of hypertension was present in 17 patients. None of the patients had a past or family history of psychiatric disorders. The mean BMI of the patients was 25.6 (±2.5) kg/m^2^. The BMI ranged from 20.9 kg/m^2^ to 30.2 kg/m^2^. The mean level of serum cholesterol was 164.9 (+10.3) mg%. The values of serum cholesterol ranged from 140 to 180 mg%. Hypertension was present in 23 patients. 

The results of the psychological tests given to the patients before and after PTCA are shown in Table 2. On the HADS, 26 (46.4%) patients had definite anxiety before PTCA, but none had definite anxiety after PTCA. On the other hand, before PTCA, 18 (32.1%) patients had definite depression, and even after the successful PTCA, 2 (3.6%) patients continued to have definite depression.

 None of the patients suffered any complications following the procedures, which were all successful. The mean duration of stay in CCU was 1.2 (±0.4) days and ranged from 1 to 2 days. The mean duration of hospitalization was 3.6 (±0.9) days and ranged from 2 to 5 days.

## 4. Discussion

 Contemporary cardiology excels at managing life-threatening conditions (e.g., acute MI and unstable angina) with high-tech methods for diagnosis and treatment such as the coronary care unit, thrombolysis, PTCA, and CABG surgery. However, cardiologists have generally been far less interested in the day-to-day support that is often needed to help patients maintain a heart-healthy lifestyle and thus reduce the risk for cardiac events. It is here that mental health practitioners may find their expertise of great benefit, particularly for individuals who develop CHD before the age of 65, when the disease is considered premature and often has a substantial lifestyle component. In the present study, we found that over 50% of the patients were below 65 years of age and therefore could benefit from psychological intervention. Psychological stress, Type A behavior pattern, anger and hostility, and social isolation or lack of social support are some of the psychosocial variables that have linked to the development of CHD in otherwise healthy populations. In addition, depression has been shown to increase CHD risk and mortality after myocardial infarction (MI). Other psychosocial variables that may contribute to CHD risk include job strain, vital exhaustion (lack of energy, demoralization, and increased irritability), anxiety, and cardiac denial (ignoring the significance of cardiac symptoms). Psychological intervention with cardiac patients reduces psychological pain—severe anxiety, hostility, and depression—and thus improves quality of life as well. In my experience, successful psychological treatment of cardiac patients has resulted in more satisfying lives, not only for patients but also for their families. The most compelling data in behavioral medicine today are to be found in cardiac psychology. There is a large database linking behavioral and psychological factors with the onset of CAD as well as with secondary cardiac events in individuals who already have CAD. This direction seems particularly relevant in light of the current economic climate promoting reduced medical costs through prevention [11–13].

The present study also revealed that patients did have modifiable factors like having high BMI, being nonvegetarian and smokers, hypertension, and increased plasma cholesterol levels. In addition, psychological evaluation revealed that 46% of the patients had definite anxiety before PTCA. This may be indicative of the stress before the procedure because three days after PTCA none of the patients had definite anxiety. Obviously, some psychological intervention is indicated before PTCA. Similarly, it was seen that 18 (32.1%) patients had definite depression and even after the successful PTCA 2 (3.6%) patients continued to have definite depression. It is therefore imperative that depression in PTCA patients should be identified and treated since this may improve morbidity and mortality [14].

 Though the numbers of patients in the present study were only 35 and the number of patients with anxiety and depression is very small, this finding is important. Depression has long been linked to poor medical compliance, to other risk factors for CAD such as smoking and obesity, and to greater functional impairment. Depression also independently predicts the development of CAD in the general population, as well as future cardiac events and mortality in patients with CAD. Anxiety or anxiety disorders independently predict sudden cardiac death in the general population as well as future cardiac events in patients with CAD [15]. In an earlier study, survival in 222 low-risk male patients 7 years after the first MI was predicted by the following psychosocial variables: lack of partnership, depression, and anxiety. Although it is unknown whether the risk of dying 7 years after an initial MI can be reduced by therapeutic interventions, these data reinforce the importance of special attention for patients with these psychosocial characteristics [16]. The findings of the present study emphasize the importance of screening patients selected for cardiac intervention for anxiety and depression because this not only improves the quality of life but also may reduce morbidity and mortality after the intervention. 

Neuroticism has been cited as a risk factor for IHD, and the Coronary Scale was constructed with the aim of measuring it. However, in the present group of patients with IHD, all the patients scored less than the cutoff score. Thus, the Coronary Scale does not seem to be useful in our population to measure proneness to IHD. Of course, the present sample consisted of only males who were serving or retired armed forces personnels. Therefore, further studies with different populations are indicated. 

On the Seattle angina questionnaire there was a significant reduction in Physical limitation from 67.9 (8.8) to 48.0 (12.4). In addition there was a significant improvement in Treatment satisfaction from 39.4 (5.7) to 61.6 (4.7). Both these findings indicate that the PTCA was successful.

Outcome after invasive treatment for CAD is assessed in terms of major adverse cardiovascular events, that is, in-hospital mortality, cardiovascular complications, and long-term survival. However, outcome cannot be assessed solely in objective terms, as such indicators do not explain how people perceive and experience their own lives. This has led to assessing outcome also in terms of patients' perception of changes in their state of health over time and how this affects their lives by assessing the health related quality of life (HRQOL). Improvement of HRQOL is associated with better prognosis [17, 18]. In the present study, we found that there was a significant improvement of HRQOL from 42.7 (15.3) to 78.7 (8.3). In addition, there was also a nonsignificant improvement in mobility, self-care, usual activities, psychological functioning, and reduction in pain. This was probably due to the fact that in the present study all, the patients had successful PTCA. It shows that the EQ5D is a useful test that can be used in PTCA patients. 

The findings of the present study emphasize the importance of the role of mental health professional in the comprehensive care of patients with CHD. The mental health professional working in a cardiac setting will undoubtedly work with patients who vary widely in their knowledge about CHD. Generally, the more thoroughly the patient understands the disease, the greater the opportunity to focus on psychological issues is, and therefore knowledge about CHD needs to be imparted to the patients. After an initial interview or a few individual sessions, group therapy is often the treatment of choice. Patients enjoy the camaraderie and benefit from their shared experience in managing risk factors and dealing with the emotional effects of this chronic, generally progressive disease.

Study limitations include the relatively low number of subjects due to time constraints. Observer rated instruments are superior to self-rated forms, but psychiatric evaluation of all the patients in CCU was not possible. However, the use of standardized measures probably reduced this limitation.

## 5. Conclusion

Successful PTCA in patients with CAD resulted in significant reduction in anxiety, depression, physical limitation and significant improvement in disease perception and health status soon after the procedure.

## Figures and Tables

**Table 1:** Demographic characteristics and habits of the patients undergoing PTCA.

Characteristics	No. of patients	Percentage
Age distribution		
20–29 years	1	2.9
30–39 years	1	2.9
40–49 years	0	0
50–59 years	14	40
60–69 years	12	34.3
70–79 years	5	14.3
80–89 years	2	5.7
Marital status		
Married	29	82.9
Widower	6	17.1
Education		
Up to 10 classes	19	54.3
11-12 classes	8	22.9
Graduates	8	22.9
Family income/month		
Rs 5000–Rs 7500	16	45.7
Rs 7501–Rs 1000	19	54.3
Food habits		
Vegetarian	15	42.9
Nonvegetarian	20	57.1
Smoking		
Nonsmoker	16	45.7
<10 cigarettes/day	5	14.3
10–19 cigarettes/day	13	37.1
20/more cigarettes/day	1	2.9
Alcohol		
Nondrinker	22	62.9
Occasional drinker	1	2.9
1 peg/week	1	2.9
1 peg/day	11	31.4

**Table 2:** Psychological test results of the patients undergoing PTCA.

Test	Pre-PTCA score	Post-PTCA score	Wilcoxon test
	Mean (±SD)	Mean (±SD)	
The Hospital Anxiety and Depression Scale			
Anxiety	12.7 (2.3)	6.1 (1.5)	S
Depression	11.4 (2.7)	7.2 (1.9)	S
Coronary scale			
Neuroticism	2.9 (1.6)	—	
Seattle Angina Questionnaire			
Physical limitation	67.9 (8.8)	48.0 (12.4)	S
Angina stability	9.6 (12.3)	7.9 (11.1)	NS
Angina frequency	9.8 (9.3)	7.0 (9.9)	NS
Treatment satisfaction	39.4 (5.7)	61.6 (4.7)	S
Disease perception	21.2 (4.7)	37.1 (5.1)	S
EQ5D Health Questionnaire			
Mobility	1.5 (0.6)	1.1 (0.4)	NS
Self-care	1.4 (0.5)	1.03 (0.17)	NS
Usual activities	1.4 (0.6)	1.1 (0.26)	NS
Pain	1.97 (0.6)	1.1 (0.5)	NS
Psychological functioning	1.8 (0.7)	1.3 (0.7)	NS
Health status	42.7 (15.3)	78.7 (8.3)	S

S: significant; NS: not significant.
